# Indole-3-Propionic Acid as a Potential Therapeutic Agent for Sepsis-Induced Gut Microbiota Disturbance

**DOI:** 10.1128/spectrum.00125-22

**Published:** 2022-06-06

**Authors:** Heng Fang, Miaoxian Fang, Yirong Wang, Huidan Zhang, Jiaxin Li, Jingchun Chen, Qingrui Wu, Linling He, Jing Xu, Jia Deng, Mengting Liu, Yiyu Deng, Chunbo Chen

**Affiliations:** a Department of Critical Care Medicine, Guangdong Provincial People’s Hospital, Guangdong Academy of Medical Sciences, Guangzhou, Guangdong, China; b Department of Intensive Care Unit of Cardiac Surgery, Guangdong Cardiovascular Institute, Guangdong Provincial People’s Hospital, Guangdong Academy of Medical Sciences, Guangzhou, Guangdong, China; c The Second School of Clinical Medicine, Southern Medical University, Guangzhou, Guangdong, China; d Clinical Research Center, Maoming People’s Hospital, Maoming, Guangdong, China; Nanjing Agricultural University

**Keywords:** sepsis, gut microbiota, indole-3-propionic acid, microbiota metabolite

## Abstract

The effects of using gut microbiota metabolites instead of live microorganisms to modulate sepsis-induced gut dysbiosis remain largely unknown. We assessed the effects of microbiota metabolite indole-3-propionic acid (IPA) on gut microbiota in mice during sepsis. Sepsis models were constructed by cecal ligation and puncture (CLP) methods. Fecal microbiota composition analysis was performed to characterize the gut microbiota composition. Fecal microbiota transplantation was performed to validate the roles of gut microbiota on sepsis progression. IPA-treated mice exhibited lower serum inflammatory mediator levels and a higher survival rate than those of saline-treated mice after modeling of sepsis, which were negated in the presence of antibiotics. Compared with saline-treated mice after modeling, IPA-treated mice showed a markedly different intestinal microbiota composition, with an enrichment of *Bifidobacteriaceae* family and a depletion of *Enterobacteriaceae* family. Mice gavaged with postoperative feces from IPA-treated animals displayed better survival than mice gavaged with feces from saline-treated animals. Overall, these data suggest that IPA offers a microbe-modulated survival advantage in septic mice, indicating that some microbiota metabolites could replace live microorganisms as potential options for regulation of sepsis-induced gut dysbiosis.

**IMPORTANCE** The role of gut microbiota in the pathophysiology of sepsis is gaining increasing attention and developing effective and safe sepsis therapies targeting intestinal microorganisms is promising. Given the safety of probiotic supplementation or fecal microbiota transplantation in critically ill patients, identifying an abiotic agent to regulate the intestinal microbiota of septic patients is of clinical significance. This study revealed that IPA, a microbiota-generated tryptophan metabolite, ameliorated sepsis-induced mortality and decreased the serum levels of proinflammatory cytokines by modulating intestinal microbiota. Although IPA did not increase the abundance and diversity of the microbiota of septic mice, it significantly decreased the number of *Enterobacteriaceae* family. These findings indicate that a specific microbiota metabolite (e.g., IPA) can mediate the intestinal microbiota apart from FMT or probiotics.

## INTRODUCTION

Sepsis is life-threatening organ dysfunction that results from a dysregulated host response to infection and has attracted increasing attention because of its high morbidity and mortality rates (1, 2). An estimated 50 million people worldwide are impacted by sepsis each year and 11 million individuals succumb to the disease (3). Given the dangers of sepsis to human health, developing effective and safe sepsis prevention and treatment strategies is critical.

Recent studies have demonstrated that the gut microbiota is severely disturbed in septic patients, which could affect treatment outcomes (4). In addition, the disrupted composition and function of gut microbiota during sepsis contribute to sepsis-linked organ dysfunction (5). Modulation of the gut microbiota through fecal microbiota transplantation (FMT) or probiotic supplementation has shown beneficial effects for septic patients in several studies (6, 7). However, FMT-transmitted microorganisms can lead to adverse drug-resistant bacteria infections, and these issues should not be ignored (8). In addition, probiotic supplementation may increase the risk of bacteremia in intensive care unit patients from probiotic bacteria transmission to the bloodstream (9). The safety of FMT and probiotics administration in the clinical setting thus remains controversial (7). Therefore, identifying an abiotic agent to regulate the intestinal microbiota of septic patients is critical and of clinical significance.

Microbial metabolites are increasingly recognized as key mediators of the injury responses in multiple organs distant from the gut (10–13). For example, gut microbiota-generated granisetron mediates the susceptibility of sepsis-induced liver injury (14), and short-chain fatty acids exert neuroprotective effects against sepsis-induced behavioral impairment (15). However, few studies have examined the effects of microbial metabolites on gut microbes and the role of these effects in sepsis progression. Nevertheless, some evidence has suggested that microbiota metabolites may regulate the composition of intestinal microbiota. For example, previous studies showed an inhibition of *Staphylococcal* growth by penicillin (16) and the inhibition of pathogenic bacterial growth by the acidic environment in the vagina maintained by *Lactobacillus* (17). Therefore, strategies using microbiota metabolites may provide a novel approach to modulate the sepsis-induced gut dysbiosis.

We speculated that intestinal microbiota metabolites may regulate the composition of microbiota and play a key role in sepsis development. A previous study reported that indole-3-propionic acid (IPA), a microbiota-derived tryptophan metabolite, inhibits gut dysbiosis in rats fed a high-fat diet (18). In addition, IPA has been reported to modulate the Escherichia coli tryptophan operon, suggesting that IPA has an effect on certain bacterial metabolic functions (19). And IPA could induce apoptosis-like death in Escherichia coli (20), which suggests that IPA can affect the growth of certain bacteria. Therefore, we selected IPA to investigate the effects of microbiota metabolites on gut microbiota and the role of these effects in the prognosis of sepsis. Our findings may help establish a novel microbiome-targeted therapeutic strategy for sepsis-induced gut microbiota disturbance.

## RESULTS

### IPA could modulate gut microbiota in healthy mice.

**FIG 1:**
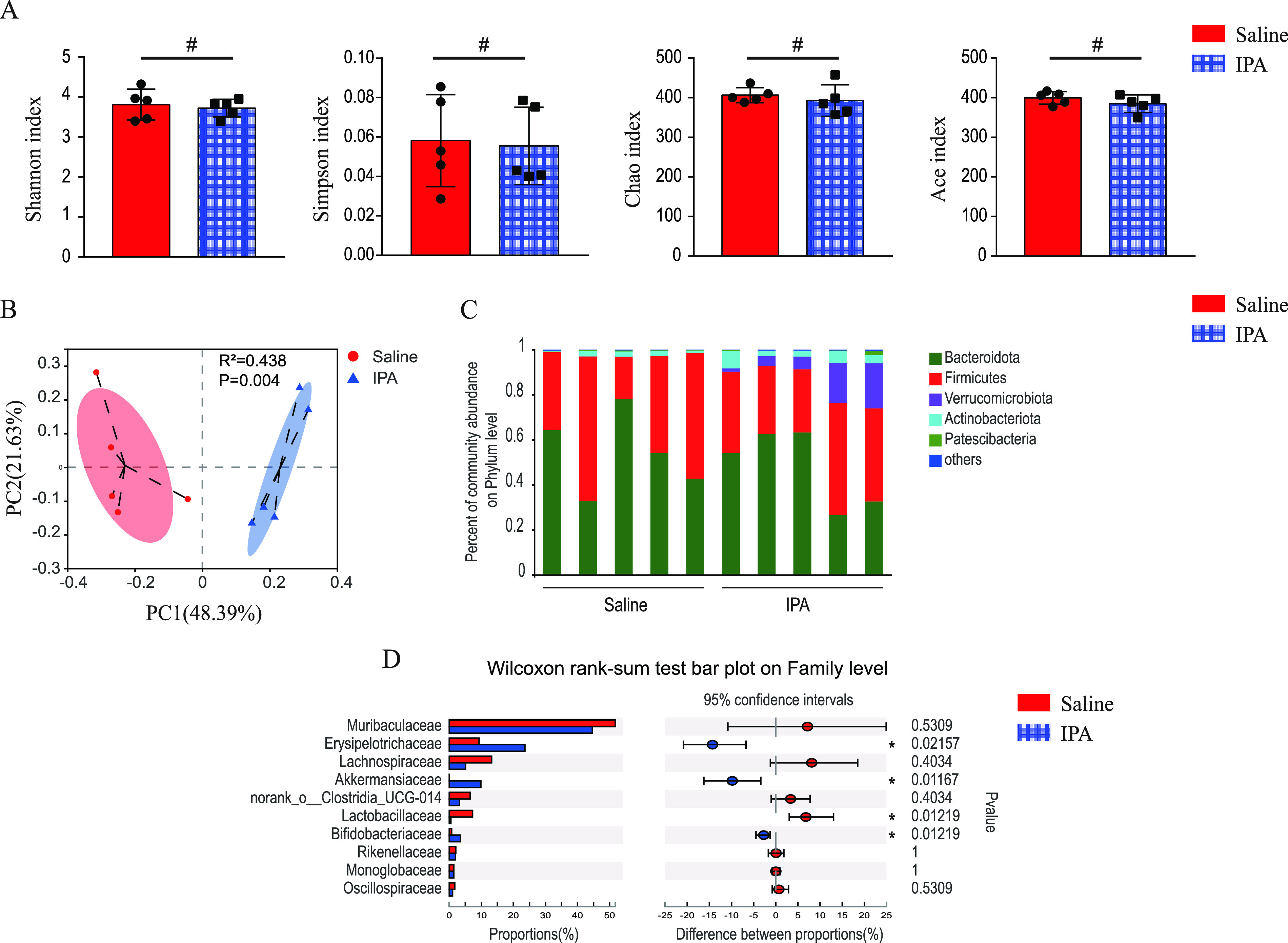
Supplementation with IPA-altered gut microbiota composition in healthy mice. (A) Composition of alpha diversity was assessed by the Shannon, Simpson, Chao, and Ace indices in feces. (B) Gut microbiota beta diversity was showed by scatterplots of PCoA. Relative abundance of phylum-level gut microbial taxa (C) and differences in the relative abundance of samples at family levels (D) were showed. *n* = 5; *, *P < *0.05; *#*, *P > *0.05.

To determine whether IPA regulates the intestinal microbiota of healthy mice, mice were gavaged with either IPA or saline for 5 days and feces were collected. We profiled the gut microbiota of animals by bacterial 16S rRNA gene sequencing. Bacterial alpha diversity (the species richness and diversity of microbiota) was evaluated by calculating Shannon, Simpson, Ace, and Chao indices in various groups, and beta diversity (between-habitat diversity) was assessed by principal co-ordinates analysis (PCoA). No significant difference in alpha diversity was observed between the two groups (Fig. 1A), indicating that IPA did not affect the bacterial richness and diversity in gut. In contrast, significant differences in beta diversity were observed between the two groups (*P* = 0.004) (Fig. 1B), which demonstrates that the IPA intervention markedly altered gut microbiota composition in healthy mice. Specific bacteria at different taxonomic levels were enriched or depleted in the IPA group compared with those in control animals (Fig. 1C and D). The samples of the IPA group were highly enriched with *Akkermansiaceae* and *Bifidobacteriaceae*, two families associated with health (21, 22). Interestingly, the saline group was enriched with the *Lactobacillaceae* family.

### IPA improved survival and reduced systemic inflammation in the sepsis model.

**FIG 2:**
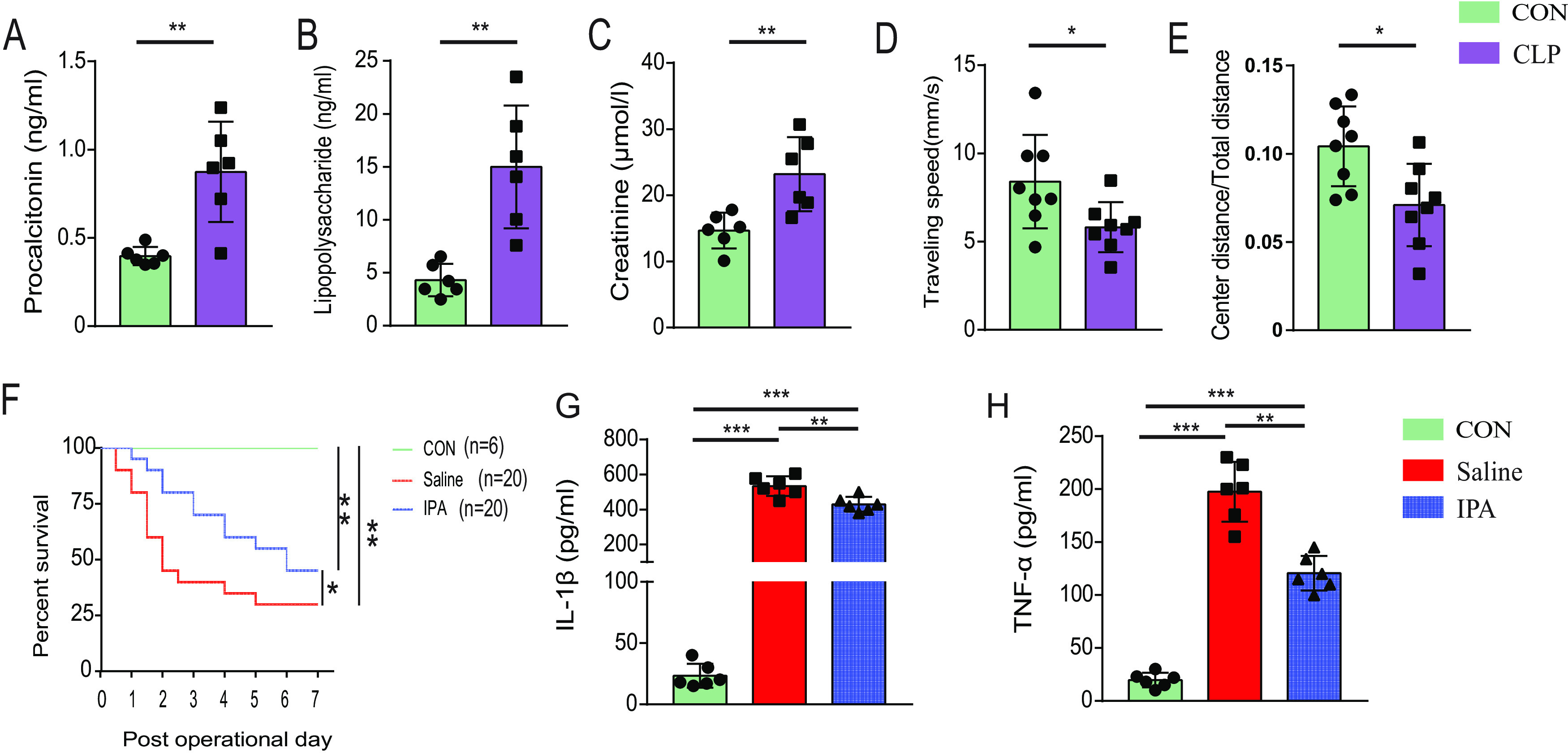
IPA increased survival and decreased serum levels of proinflammatory cytokines in mice after cecal ligation and puncture (CLP) operation. The serum levels of procalcitonin (A), lipopolysaccharide (B), and creatinine (C) were measured using enzyme-linked immunosorbent assay (ELISA) in the sham-operated (CON) and CLP-operated mice. (D, E) Mice who survived 24 h after surgery were subjected to open field test to assess their motor ability and anxiety behavior. The traveling speed (D) and the ratio of center distance to total distance (E) were collected and analyzed. (F) Survival rate. The serum levels of IL-1β (G) and TNF-α (H) were measured using ELISA in CON, IPA-treated, and saline-treated mice. *n* = 6 to 20; *, *P < *0.05; **, *P < *0.01; ***, *P < *0.001.

We next examined the effects of IPA on sepsis. Cecal ligation and puncture (CLP) was conducted to induce sepsis in mice. We found that the serum procalcitonin levels were significantly higher in the CLP-operated mice than those in the sham-operated (CON) mice at 24 h after operation (*P* = 0.002) (Fig. 2A), suggesting that the CLP-operated mice were infected. In addition, the serum Lipopolysaccharide (LPS) levels of mice after CLP operation was also significantly higher than those of sham-operated mice (*P* = 0.001) (Fig. 2B). We further explored the renal function and brain function of mice at 24 h after the operation. Regarding the renal function, serum creatinine levels were elevated in the CLP-operated mice (*P* = 0.007) (Fig. 2C). Mice who survived 24 h after the surgery were subjected to open field test to assess their anxiety behavior and motor ability. The results showed that compared with the sham-operated mice, the CLP model mice had poorer mobility (*P* = 0.029) and more obvious anxiety behaviors (*P* = 0.012) (Fig. 2D and E). Collectively, the CLP-operated mice developed sepsis in this study.

After 5 days of IPA or saline intervention, mice were subjected to CLP and survival rates were observed (Fig. 2F). Mortality was higher in CLP-operated mice compared with the sham-operated group. Among CLP-operated mice, death occurred in approximately half of the saline-treated mice within 48 h compared with 20% in the IPA group. In addition, the 7-day mortality rate in the saline group was 70% compared with 55% in the IPA group. Mice that were gavaged with IPA exhibited a reduction in overall mortality.

We then explored the effects of IPA on inflammation responses in mice at 1 day after CLP (Fig. 2G and H). Serum levels of proinflammatory cytokines interleukin (IL)-1β (*P* < 0.001) and tumor necrosis factor (TNF)-α (*P* < 0.001) were significantly increased in CLP-operated mice compared with levels in sham-operated mice. In CLP-operated mice, IPA reduced the serum concentrations of IL-1β (*P* = 0.001) and TNF-α (*P* < 0.001).

### Dependence of IPA on gut microbiota for protection against septic death.

**FIG 3:**
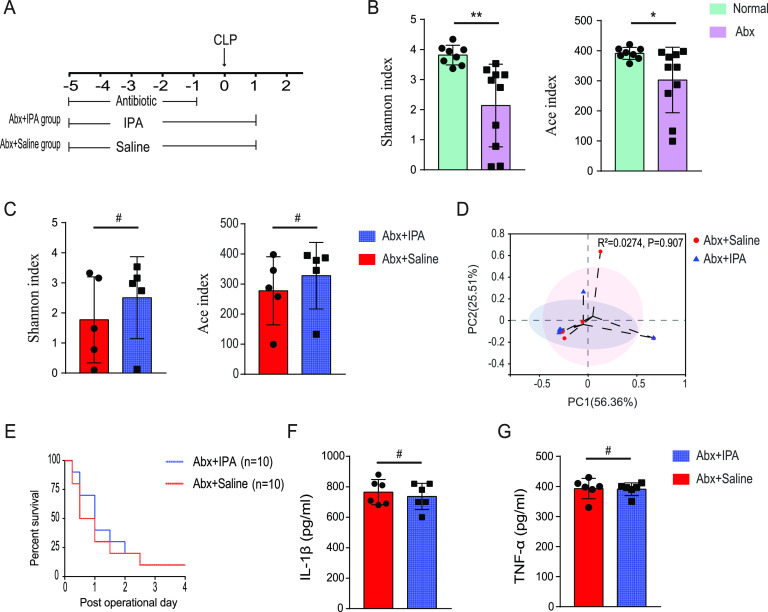
Antibiotic administration negates the protective effect of IPA supplementation. (A) The schematic diagram showed experimental design and procedures for antibiotic (Abx) administration. (B) Gut microbiota alpha diversity was assessed by the Shannon and Ace indices in antibiotic-treated and normal mice. (C) Gut microbiota alpha diversity was assessed by the Shannon and Ace indices in the IPA-treated and saline-treated mice after antibiotic administration. (D) Scatterplots of PCoA showed gut microbiota beta diversity between the IPA-treated and saline-treated mice after antibiotic administration. (E) Survival rate. The serum levels of IL-1β (F) and TNF-α (G) were measured using ELISA. *n* = 6 to 10. *#*, *P* > 0.05.

After identifying the effects of IPA on gut microbiota and sepsis progression separately, we investigated whether the protective effect of IPA against sepsis is mediated in part by the gut microbiota. In addition to supplementation with saline or IPA, animals were administered broad-spectrum oral antibiotics by gavage once daily for 5 days to deplete gut microbiota prior to CLP (Fig. 3A). The antibiotic regimen was 100 mg of vancomycin, 200 mg of metronidazole, 200 mg of neomycin sulfate, and 200 mg of ampicillin per kilogram body weight of the mice. Compared with normal mice, the abundance (*P* = 0.012) and diversity (*P* = 0.001) of gut microbiota were significantly decreased in antibiotic-treated mice (Fig. 3B). In addition, there was no significant difference in the gut microbiota abundance and diversity between the IPA-treated and saline-treated mice after the antibiotic treatment (Fig. 3C). PCoA analysis revealed that clusters of gut microbiota between the IPA-treated and saline-treated mice were not completely separated after the antibiotic treatment (Fig. 3D), indicating that the gut microbiota exhibited a similar composition in IPA-treated and saline-treated mice in the presence of antibiotics. Under the antibiotic intervention protocol, we found that IPA did not show a protective effect on mice after CLP surgery (Fig. 3E). Serum TNF-α and IL-1β levels were similar in IPA-treated and saline-treated diet groups (Fig. 3F and G). These results indicate that the survival and anti-inflammatory benefits conferred by IPA were dependent on the intestinal microbiota.

### Effects of IPA on gut microbiota composition of septic mice.

**FIG 4:**
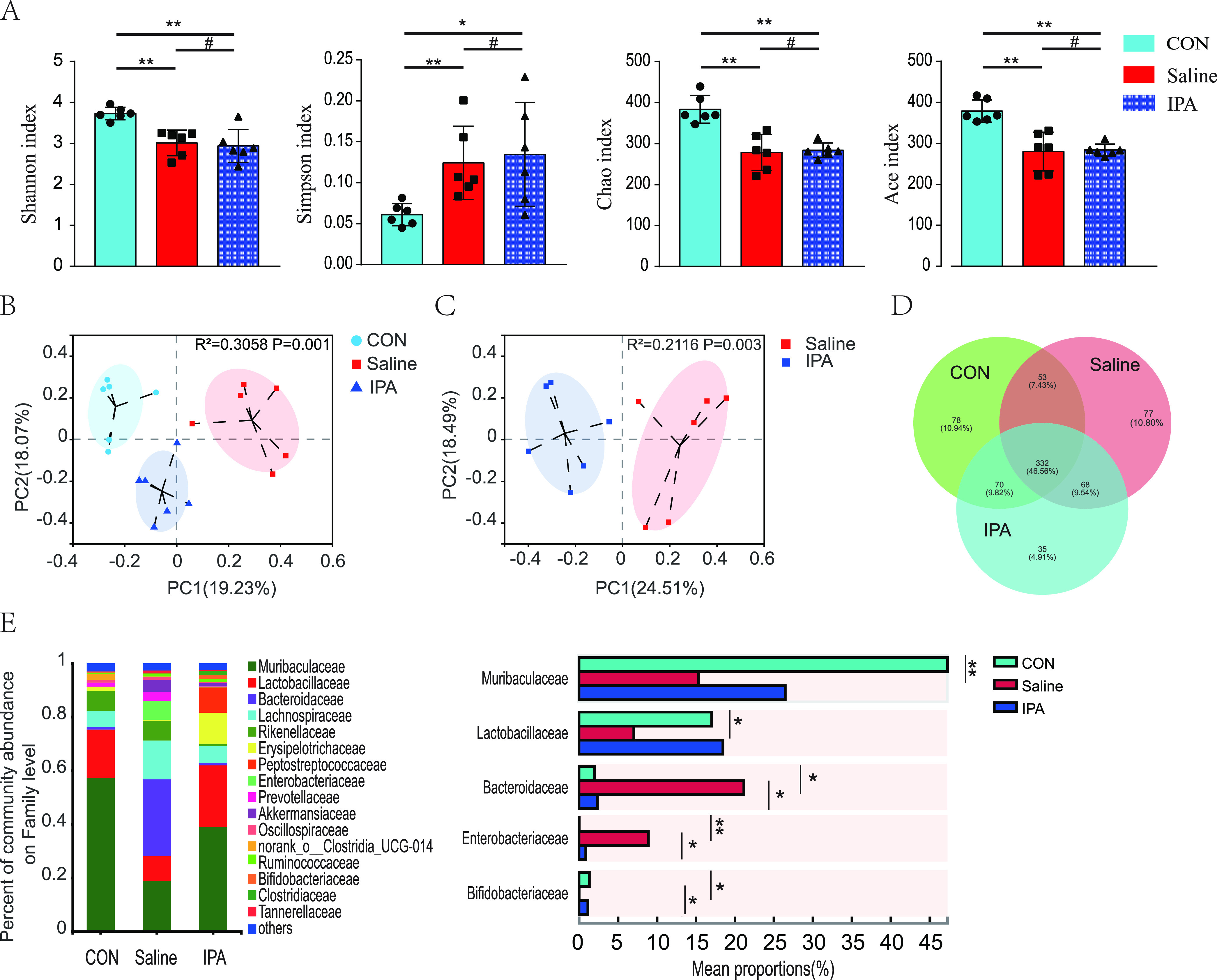
Supplementation with IPA-altered gut microbiota composition in septic mice. (A) Composition of alpha diversity was accessed by the Shannon, Simpson, Chao, and Ace indices in feces. (B, C) Scatterplots of PCoA showed beta diversity of feces. Venn diagram of the number of common and unique operational taxonomic units (D) and variation in the relative abundance of samples at the family level (E) in each group were showed. *n* = 6; *, *P < *0.05; **, *P < *0.01; ***, *P < *0.001; *#*, *P > *0.05.

Given that the protective effect against septic death provided by IPA is dependent on gut microbiota, we evaluated the alterations of the gut microbial composition in mice after CLP. We found reduced gut microbiota alpha diversity in CLP-operated mice compared with those in sham-operated animals, while no significant difference was observed in alpha diversity between IPA-treated and saline-treated groups (Fig. 4A). We also observed significant differences in beta diversity of the samples in each operated group (Fig. 4B and C). These results indicate that mice undergo a significant alteration of gut microbiota after modeling of sepsis and that IPA modulates the community composition.

There are common or unique operational taxonomic units in each group (Fig. 4D). Compared with the sham-operated mice, saline-treated animals had a lower abundance of *Muribaculaceae*, *Lactobacillaceae*, and *Bifidobacteriaceae* and a higher abundance of *Bacteroidaceae* and *Enterobacteriaceae* at the family level (Fig. 4E). *Enterobacteriaceae* is associated with poor prognosis in several diseases, including sepsis (23, 24). However, the abundance of *Bifidobacteriaceae* was increased and the abundance of *Bacteroidaceae* and *Enterobacteriaceae* was reduced in IPA-treated mice compared with levels in saline-treated animals. Together, these findings indicate that IPA modulated the gut microbiota composition during sepsis and, in particular, depleted *Enterobacteriaceae* in the mouse gut.

### Protective effect of IPA against sepsis was transmissible by gut microbiota.

**FIG 5:**
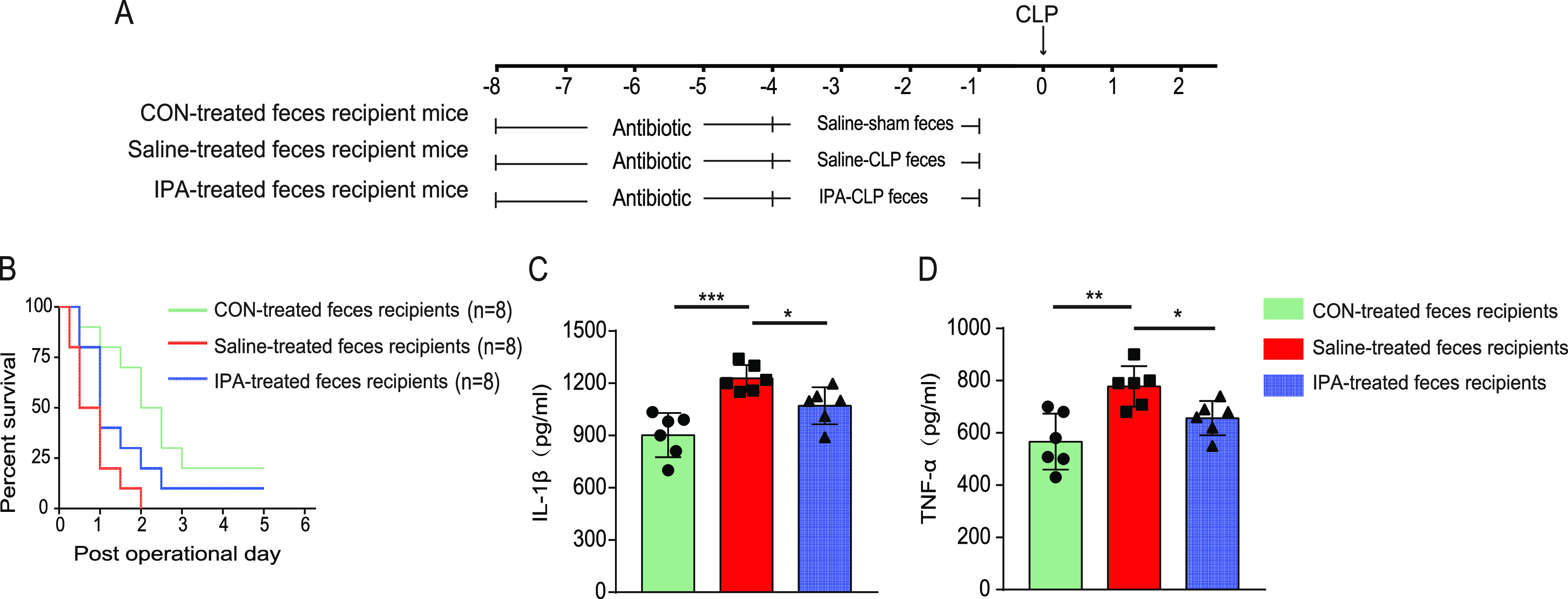
Gut microbiota composition modulated sepsis progression. (A) The schematic diagram showed the fecal microbiota transplantation (FMT) experimental design and procedure. (B) Survival rate (*n* = 8). The serum levels of IL-1β (C) and TNF-α (D) were measured using ELISA (*n* = 6). *, *P < *0.05.

Our results revealed substantial differences in the intestinal microbiota composition of the three operated groups. We thus further explored the relationship between microbiota composition and sepsis by conducting an FMT experiment (Fig. 5A). After treatment with antibiotic cocktail to deplete the intestinal microbiota for 5 days, mice received feces from sham-operated, saline-treated, or IPA-treated CLP-operated animals. All mice that received saline-treated FMT died within 48 h. Sham-operated feces recipients showed a higher 5-day survival rate and a lower level of proinflammatory cytokines (IL-1β, *P* < 0.001; TNF-α, *P* = 0.001) than saline-treated feces recipients (Fig. 5B to D), indicating that sepsis-induced gut dysbiosis exacerbates sepsis. In addition, recipients of IPA-treated feces showed a higher 5-day survival rate and a lower level of proinflammatory cytokines (IL-1β, *P* < 0.021; TNF-α, *P* = 0.026) than those of saline-treated feces recipients. These findings suggest that the protective effect of IPA against sepsis was transferrable by gut microbiota.

## DISCUSSION

Growing evidence has demonstrated the crucial role of the gut microbiota in sepsis pathophysiology and sepsis-related organ dysfunction (4). These findings have indicated the potential of developing effective and safe sepsis therapies targeting intestinal microorganisms. This study revealed that IPA, a microbiota-generated tryptophan metabolite, ameliorated sepsis-induced mortality and decreased the serum levels of proinflammatory cytokines by modulating intestinal microbiota. To the best of our knowledge, this is the first report of the effects of gut metabolites on the gut microbiota during sepsis, supporting the general concept that gut microbiota modulates sepsis progression. More importantly, we could use a specific microbiota metabolite (e.g., IPA) to mediate the intestinal microbiota rather than just choosing from FMT, probiotics, or prebiotics.

The transfer of live microorganisms from healthy donors to recipients via FMT, or the direct use of “good” bacteria such as *Lactobacillus*, *Bifidobacterium*, and Streptococcus, rescues disrupted microbial ecosystems (6). Despite the successful results from clinical studies (25–27), the risk of additional bacteremia transmission in critically ill patients remains a concern (7). In this study, it was found that IPA significantly altered the intestinal microbiota composition of untreated mice, without supplementation with live microorganisms, and did not reduce the species richness and diversity of microbiota within 5 days (Fig. 1A). These results confirmed that IPA may be effective to regulate intestinal microbiota. Interestingly, IPA significantly increased the relative abundance of *Bifidobacteriaceae*, a family that contains probiotic *Bifidobacterium*. Previous studies showed that the decline of *Bifidobacterium* in gut was associated with poor outcomes of critical patients (28), while *Bifidobacterium* expansion may protect mice from infection (29), suggesting that IPA may exert beneficial effects on the host by promoting the growth of *Bifidobacterium*.

The beneficial effects of IPA on multiple diseases such as steatohepatitis, hyperlipidemia, and diabetes have been demonstrated (18, 30, 31). IPA also modulates astrocyte activation and neuroinflammation and inhibits neuronal death induced by endoplasmic reticulum stress (32). Supplementation with IPA reduced neuroinflammation in mice with encephalomyelitis (33). Our study found that IPA reduced serum proinflammatory cytokine levels and ameliorated mortality in CLP-operated mice. These findings have enriched the understanding that IPA plays a protective role in sepsis pathophysiology. However, in the present study, IPA did not improve the prognosis of septic mice with intestinal microbiota depleted by antibiotics, suggesting that the protective effects of IPA are gut microbiota-dependent.

We found that the intestinal microbiota of septic mice was severely disrupted, with a marked decrease in richness and diversity and a marked increase in the abundance of opportunistic pathogenic bacteria such as *Enterobacteriaceae*, which is consistent with previous reports (24, 34). Gut *Enterobacteriaceae* expansion is considered a detrimental factor in patient prognosis (23, 24). *Enterobacteriaceae* overgrowth in the gut could exacerbate brain infarction and systemic inflammation of stroke patients (23). Moreover, *Enterobacteriaceae* expansion in the gut significantly raises the risk of bloodstream invasion, sepsis, and death (35). Our data showed that although IPA did not increase the abundance and diversity of the microbiota of septic mice, it significantly decreased the number of *Enterobacteria*ceae, indicating that IPA ameliorates mortality from sepsis by inhibiting the overgrowth of *Enterobacteriaceae* in the gut.

Several reasons may explain the inhibition effect of IPA on *Enterobacteriaceae* in gut. One possible reason is that IPA may have a direct inhibitory effect on *Enterobacteriaceae*. IPA exhibits antibacterial activity in Escherichia coli of the *Enterobacteriaceae* family, and the mechanism may involve induction of the accumulation of reactive oxygen species, nitric oxide, peroxynitrite, and DNA damage, ultimately leading to apoptosis-like death in E. coli (20). Another possible reason is that IPA increases the abundance of *Bifidobacteriaceae*, and *Bifidobacterium* inhibits the growth of pathogenic bacteria (21). We further explored the effects of variability in sepsis-induced gut dysbiosis influenced by IPA on the sepsis progression in FMT experiments. The results suggested that gut dysbiosis characterized by *Enterobacteriaceae* enrichment may increase serum proinflammatory cytokine levels and worsen the sepsis prognosis, which enriches the evidence for the harmful role of *Enterobacteriaceae* in sepsis (35).

Numerous studies have shown that pathogen infection leads to activation of the host innate immunity, followed by a “cytokine storm” that creates a state of immune dysregulation (36). An excessive proinflammatory response and/or exaggerated anti-inflammatory response further induce cellular dysfunction and death, ultimately resulting in multiorgan dysfunction syndrome and death (36). Similar to other studies (37, 38), we also found that serum levels of the proinflammatory cytokines IL-1β and TNF-α were significantly elevated in septic mice (Fig. 2G and H). In addition, IPA reduced the serum concentration of proinflammatory cytokines in septic mice, suggesting the anti-inflammatory activity of IPA. However, IPA did not influence the serum levels of IL-1β and TNF-α in antibiotic-treated septic mice, suggesting that the anti-inflammatory activity of IPA was possibly associated with intestinal microbiota (Fig. 3F and G). There may be two reasons why IPA did not reverse sepsis after antibiotic treatment. One is the effect of the antibiotics themselves on the mice. Antibiotics have been reported to differentially modulate lipoteichoic acid-mediated host immune response (39). And in this study, we used nonphysiologic doses of antibiotics, which may have affected the mice’s response to sepsis. The other one is that antibiotics modulate the mice's response to sepsis by affecting the gut microbiota. The gut microbiota has been found to interact with the host immune response against pathogens (40). In addition, In the present study, recipients of IPA-treated feces showed a higher 5-day survival rate and a lower level of proinflammatory cytokines than those of saline-treated feces recipients (Fig. 5B to D), which also supports that the anti-inflammatory activity of IPA is regulated by the gut microbiota.

A possible reason for the association of IPA’s anti-inflammatory activity with intestinal microbiota is that IPA increased the abundance of *Bifidobacteriaceae* and inhibited the expansion of *Enterobacteriaceae*, both of which were found to have an impact on the host immune system (41, 42). In addition, IPA was found to regulate certain bacterial metabolic functions (19) and gut microbiota-generated metabolites have been reported to be involved in host inflammatory responses (43). The effect of IPA on the production of gut microbiota metabolites may be another reason for the IPA’s anti-inflammatory activity through gut microbiota.

Despite the growing interest in the effects of microbial metabolites on multiple diseases (44), the precise effects of microbial metabolites on gut microbiota in disease progression remain to be elucidated. Our studies demonstrated that gut microbiota-produced IPA regulated the gut microbiota composition of septic mice, thereby protecting against sepsis. We found that the *Enterobacteriaceae* expansion in the gut of septic mice was significantly inhibited by IPA and the mechanism underlying how IPA suppresses the bacteria overgrowth needs to be further explored. Our study showed that supplementation with a specific microbial metabolite, in addition to FMT and probiotics, is a potential therapeutic approach targeting the microbiome in the management of sepsis. These findings may have potential translational value for future clinical management of septic patients.

### Conclusion.

By using a gut microbiota-produced metabolite, IPA, as a microbiome regulatory compound, our study found that certain microbial metabolites may function by modulating the intestinal microbiota of mice during sepsis. Administration of IPA targeting the microbiome may provide a new potential approach for the clinical management of sepsis.

## MATERIALS AND METHODS

### Animal experimental design.

All animal studies were approved by the local Animal Care and Use Committee of Guangdong Provincial People’s Hospital (approval #GDREC2018199A). A sepsis model was induced in male C57BL/6 mice (6 to 8 weeks old) by cecum ligation and puncture (CLP), as described previously (14). Briefly, mice were anesthetized with pentobarbital and CLP was conducted under sterile conditions. A midline incision of approximately 1 cm was made in the abdomen and the cecum was exposed. The cecum was ligated at the center and punctured with a 21-gauge needle. A small amount of feces was extruded through the puncture, and then the cecum was gently repositioned in the peritoneal cavity. The incision was then sutured. Sham controls (CON group) underwent laparotomy and manipulation to expose the cecum without ligation and puncture. IPA-treated mice received IPA (Sigma catalog number 220027) by gavage (25 mg/kg dissolved in saline) once daily for 5 days before and once within 1 day after establishment of the model, following a modified treatment protocol based on previous studies (33, 45) and our pilot trials. Briefly, after treatment with antibiotic cocktail for 5 days to deplete the gut microbiota, mice received feces from donor mice for 3 days. Saline-treated mice received equal amounts of saline administration. Mouse feces were collected 1 day before and 2 days after the CLP operation and stored at –80°C. Serum was collected 24 h after surgery.

### Fecal microbiota composition analysis.

Stool microbial DNA was extracted using the Mag-Bind Soil DNA kit (Omega Bio-Tek, USA) according to the manufacturer’s protocols. Then, 16S rRNA amplicons were performed on the V3–V4 hypervariable region with primer pairs 338F (5′-ACTCCTACGGGAGGCAGCAG-3′) and 806R (5′-GGACTACHVGGGTWTCTAAT-3′). Sequencing was conducted using Illumina’s Miseq PE300 platform (Illumina, San Diego, CA, USA) by Majorbio (Shanghai, China). Sequences with operational taxonomic units and chimeras were removed at 97% similarity level using UPARSE software (version 7.1). Mothur and QIIME2.0 software were used for bioinformatics analyses. Alpha diversity was evaluated using Shannon, Simpson, Chao, and ACE indices. The beta diversity was analyzed using PCoA based on Bray-Curtis distance and Adonis analysis was used to test the *P* value. Comparison of sample composition and analysis of differences in specific communities between groups using microbial taxonomy (46) was used.

### Open field test.

Mice who survived 24 h after surgery were subjected to open field test to assess their anxiety behavior and motor ability. Each mouse was gently placed at the center of a square area (60 cm × 60 cm × 60 cm) and then allowed to explore for 5 min. The trajectory of mouse activities was recorded using a video tracking system. After each test, the field was cleaned with 75% alcohol to remove odor disturbance.

### FMT.

FMT was performed following a modified method described elsewhere (14). Mice were gavaged with antibiotics to deplete the gut microbiota. The antibiotic regimen was 100 mg of vancomycin, 200 mg of metronidazole, 200 mg of neomycin sulfate, and 200 mg of ampicillin per kilogram body weight of the mice, administered by gavage once a day for 5 days. To investigate the effect of IPA-mediated gut microbiota on sepsis progression, mice were randomly divided into three groups (control feces, saline-treated feces, and IPA-treated feces recipient groups). After antibiotic treatment, mice received postoperative feces from donor mice. For the FMT protocol, donor feces were resuspended in saline at a concentration of 0.125 g/mL and 0.15 mL of the suspension was administered to mice by oral gavage once daily for 3 days.

### Enzyme-linked immunosorbent assay.

The serum levels of TNF-α, IL-1β, procalcitonin, and lipopolysaccharide were measured by enzyme-linked immunosorbent assay (ELISA) kits (Cusabio) according to the manufacturer’s instructions. Serum levels of creatinine were measured by ELISA kit (MEIMIAN).

### Statistical analyses.

Statistical analyses were performed using SPSS 20.0. All the experimental data were shown as the mean ± standard deviation (SD). Unpaired Student’s *t* test (normally distributed) or Mann–Whitney U test (not normally distributed) were used to assess between-group differences. Multiple comparison between groups using the ANOVA and LSD test. Post-hoc analysis was performed for the ANOVA testing. *P* values <0.05 were considered to indicate statistical significance.

### Data availability.

The data sets used and analyzed during the current study are available from the authors on reasonable request, some have already been included in this article. Raw sequence data of microbiota which support the findings in our study have been deposited in the SRA of the NCBI under accession number PRJNA788095.
